# Ribosome biogenesis is essential for hemogenic endothelial cells to generate hematopoietic stem cells

**DOI:** 10.1242/dev.202875

**Published:** 2024-10-23

**Authors:** Di Liu, Haizhen Wang, Haifeng Chen, Xitong Tian, Yuqing Jiao, Chi Wang, Yuhui Li, Zongcheng Li, Siyuan Hou, Yanli Ni, Bing Liu, Yu Lan, Jie Zhou

**Affiliations:** ^1^ Department of Neurology, Xuanwu Hospital Capital Medical University, National Center for Neurological Disorders, Beijing 100053, China; ^2^ The Fifth Affiliated Hospital of Southern Medical University, Southern Medical University, Guangzhou, Guangdong 510900, China; ^3^ Dermatology Hospital, Southern Medical University, Guangzhou 510091, China; ^4^ Chinese PLA medical school, Chinese PLA General Hospital, Beijing 100853, China; ^5^ State Key Laboratory of Experimental Hematology, Department of Hematology, Fifth Medical Center of Chinese PLA General Hospital, Beijing 100071, China; ^6^ Key Laboratory for Regenerative Medicine of Ministry of Education, Institute of Hematology, School of Medicine, Jinan University, Guangzhou, Guangdong 510632, China; ^7^ Medical Innovation Research Division of Chinese PLA General Hospital, Beijing 100853, China

**Keywords:** Hematopoietic stem cell development, Hemogenic endothelial cells, Ribosome biogenesis, Mouse

## Abstract

Undergoing endothelial-to-hematopoietic transition, a small fraction of embryonic aortic endothelial cells specializes into hemogenic endothelial cells (HECs) and eventually gives rise to hematopoietic stem cells (HSCs). Previously, we found that the activity of ribosome biogenesis (RiBi) is highly enriched in the HSC-primed HECs compared with adjacent arterial endothelial cells; however, whether RiBi is required in HECs for the generation of HSCs remains to be determined. Here, we have found that robust RiBi is markedly augmented during the endothelial-to-hematopoietic transition in mouse. Pharmacological inhibition of RiBi completely impeded the generation of HSCs in explant cultures. Moreover, disrupting RiBi selectively interrupted the HSC generation potential of HECs rather than T1 pre-HSCs, which was in line with its influence on cell cycle activity. Further investigation revealed that, upon HEC specification, the master transcription factor Runx1 dramatically bound to the loci of genes involved in RiBi, thereby facilitating this biological process. Taken together, our study provides functional evidence showing the indispensable role of RiBi in generating HSCs from HECs, providing previously unreported insights that may contribute to the improvement of HSC regeneration strategies.

## Teaser

**Summary:** The indispensable role of ribosome biogenesis in the generation of hematopoietic stem cells from hemogenic endothelial cells.

## INTRODUCTION

Hematopoietic stem cells (HSCs) have the capacity to both self-renew and generate all types of blood cells, supporting the life-long hematopoiesis of an organism. The first HSC is widely believed to be generated in the aorta–gonad–mesonephros (AGM) region though the process known as the endothelial-to-hematopoietic transition (EHT) (Medvinsky and Dzierzak, 1996; de Bruijn et al., 2002; Dzierzak and Speck, 2008; Zovein et al., 2008; Bertrand et al., 2010; Boisset et al., 2010; Kissa and Herbomel, 2010). Briefly, a small fraction of aortic endothelial cells (AECs) acquires hemogenic potential and specializes into hemogenic endothelial cells (HECs), which then orderly differentiate into pre-hematopoietic stem cells (pre-HSCs) and HSCs along a developmental trajectory (Taoudi et al., 2008; Rybtsov et al., 2011). Nevertheless, the underpinning mechanisms orchestrating HSC ontogeny remain poorly understood.

Muti-signaling pathways, such as those involving developmental signals (e.g. Notch, Tgf and Fgf), inflammatory signals (tumor necrosis factor, interleukin, interferon and lipopolysaccharide) and fluid biomechanical force signals, have been proved to participate in regulating HSC development (Adamo et al., 2009; Gering and Patient, 2010; Marcelo et al., 2013; Sawamiphak et al., 2014; Diaz et al., 2015; Gama-Norton et al., 2015; Monteiro et al., 2016). These signals could mediate the expression of key hematopoietic transcription factors, including Runx1, Gata2, Meis1 and Gata3, or interplay with them to elaborately regulate EHT (Chen et al., 2009; Fitch et al., 2012; Butko et al., 2015; Kang et al., 2018; Coulombe et al., 2023). Several cellular biology processes, such as the glycolysis to oxidative phosphorylation metabolic switch, also determine the differential potential of HECs (Oburoglu et al., 2022). Beyond this, a highly active cell cycle state is reported to be necessary for HEC specialization (Canu et al., 2020). Recently, taking advantage of single cell/low input omics technologies, the dynamic developmental path of EHT and the molecular signature of HECs have been deeply dissected in multiple dimensions (Hou et al., 2020; Zhu et al., 2020; Fadlullah et al., 2021; Lan, 2022). I particular, in our previous study, the HSC-primed HECs (CD41^−^CD43^−^CD45^−^CD31^+^CD201^+^Kit^+^CD44^+^, PK44) were precisely identified with the use of single-cell technologies. The specific molecular characteristics and biological processes of HSC-primed HECs were further disclosed, revealing their enrichment in cell cycle and ribosome biogenesis (RiBi) (Hou et al., 2020). RiBi is a highly coordinated process of assembling the ribosome complex. It begins with RNA polymerase I-dependent transcription of ribosomal DNA (rDNA) to produce 47S pre-rRNAs. These pre-rRNAs are then post-transcriptionally modified and processed, and incorporate with different ribosome proteins to assemble mature ribosome (Ni and Buszczak, 2023). Consequently, the transcription of 47S pre-rRNAs is the initial step of RiBi, the level of which could directly reflect RiBi activity. Additionally, the inhibition of RNA Polymerase I to block 47S pre-rRNAs transcription is a widely used approach to investigate the function of ribosome biogenesis (Baweja and Subudhi, 2021). Many studies have indicated the crucial role of RiBi in sustaining stem cell homeostasis and fate decisions (Zhang et al., 2013; Turi et al., 2019; Kang et al., 2021; Lv et al., 2021; Saba et al., 2021). And distinct RiBi levels are required in lineage commitment of both human and mouse hematopoiesis (Cai et al., 2015; Khajuria et al., 2018). Moreover, ribosomal protein paralogs, such as Rpl22 and Rpl22-like1 (Rpl22l1), were also reported to exhibit antagonistic functions in regulating embryonic HSC emergence (Zhang et al., 2013). Notably, one recent study showing that rRNA biosynthesis, which is the initial step of ribosome biogenesis, directly regulates the fate transition of mouse embryonic stem cells by maintaining the nucleolar integrity and 3D chromatins configurations (Yu et al., 2021). In addition, RiBi is also involved in regulating cell death, metabolism and cell cycle progress that relies on different cell contexts (Prakash et al., 2019; Kang et al., 2021). However, the exact roles and mechanisms underlying RiBi in regulating HSC ontogeny have yet to be elucidated.

In this study, we found that robust RiBi is significantly enriched in HECs along the HSC ontogeny. Using extensive bioinformatic and functional analyses, we have corroborated the essential role of RiBi in the generation of HSCs from HECs. Consequently, our study provides a new regulatory mechanism underlying HSC generation and offers a theoretical perspective for guiding *in vitro* regeneration of HSCs.

## RESULTS

### Dynamic changes of ribosome biosynthesis during HSC development

**Fig. 1.**
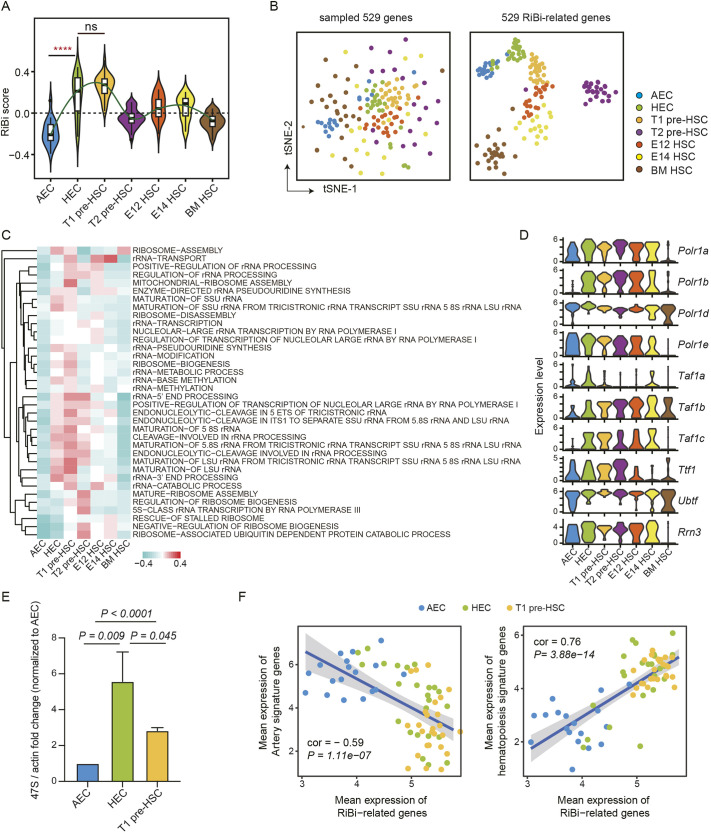
**scRNA-seq reveals dynamic change of RiBi during HSC development.** (A) Violin plot depicting the dynamic changes in RiBi scores throughout the entire HSC development. The relative activity level of RiBi is indicated by the RiBi score obtained through gene set variation analysis (GSVA) in a given set. The green curve represents the trend of change in RiBi scores and was fitted using loess regression. A Wilcoxon rank sum test is used to evaluate the significance of the differences; *P* values indicate the statistical significance (*P*<0.05 is considered statistically significant; ****P<0.0001). (B) t-SNE graphs based on different gene sets with mapped cell clusters. (C) Heatmap with average agglomerative clustering displaying GSVA scores of RiBi-related terms across all cell clusters. (D) Violin plots showing the expression levels of RNA polymerase I-related genes in seven cell clusters. The expression level is calculated by log2(TPM+1). (E) qRT-PCR detecting the expression level of 47S pre-rRNA in sorted E10.0 caudal half AECs (CD41^−^CD43^−^CD45^−^CD31^+^CD201^−^Kit^−^CD44^+^), HECs (CD41^−^CD43^−^CD45^−^CD31^+^CD201^+^Kit^+^CD44^+^) and E11.0 AGM T1 pre-HSCs (CD31^+^CD45^−^CD41^low^Kit^+^CD201^high^). Data are collected from three independent experiments. Statistical differences were assessed using an unpaired two-tailed Student's *t*-test. *P*<0.05 is regarded as statistically significant. (F) Correlation between artery and hematopoiesis signature genes, and RiBi-related genes. The colors of the points indicate the cell clusters, while the linear fit is depicted as a blue line, with the confidence interval represented by a gray area. The Pearson correlation coefficient is used to assess the correlation between the two. Absolute value of correlation coefficient >0.5 and *P*<0.05 are considered indicative of a strong correlation.

Our previous studies indicated that RiBi was enriched both in human and mouse HECs (Zeng et al., 2019; Hou et al., 2020). To substantiate these findings, we used our single-cell RNA transcriptome data (Zhou et al., 2016; Wang et al., 2022) and conducted gene set enrichment analysis (GSEA). The results reaffirmed the substantial enrichment of RiBi-related terms in both mouse and human HECs when compared with AECs (Fig. S1A). Subsequently, using a total of 529 genes associated with RiBi, we investigated their expression profiles throughout the entire HSC ontogeny in mice. We found that the RiBi score was dramatically elevated during the differentiation of AECs into T1 pre-HSCs, then gradually decreased (Fig. 1A; Table S1). In contrast to a random selection of 529 genes, it is noteworthy that these RiBi genes were able to effectively distinguish consecutive developmental stages. Additionally, several of these genes, including ribosomal subunit genes and assembly factors, displayed developmental stage-specific expression patterns, indicating the acquirement of distinct RiBi features during HSC development (Fig. 1B, Fig. S1B-D). Here, T2 pre-HSCs diverged from consecutive developmental stages along the tSNE-1 axis (Fig. 1B), showing reduced expression of ribosomal protein subunits and diminished ribosome assembly activity (Fig. S1D,H). Nevertheless, several mitochondrial ribosome-related genes were highly enriched in T2 pre-HSCs (Fig. S1B). Thus, we speculate that T2 pre-HSCs possess an overall low RiBi activity (Fig. 1A), while specifically having increased activity in mitochondrial ribosome synthesis compared with other populations. This represents a transitional cell fate that is distinct from other cells. Further functional annotation of these RiBi genes unveiled that a large number of rRNA biosynthesis-related terms were prominently enriched in HECs and pre-HSCs, encompassing rRNA transcription, rRNA modification and ribosome assembly (Fig. 1C, Fig. S1E-H). Meanwhile, there was a notable upregulation in the expression levels of RNA polymerase I constituents responsible for rRNA synthesis in both HECs and pre-HSCs, which was in harmony with the amplified transcription of the 47S rRNA precursor (47S pre-rRNA), as evidenced by quantitative PCR assays (Fig. 1D,E; Table S1).

Moreover, the expression of these RiBi-related genes positively correlated with hematopoietic feature genes, whereas it negatively correlated with genes featuring arterial endothelial identity during AEC to pre-HSCs transition (Fig. 1F, Fig. S1I). Collectively, these results indicate that RiBi is notably activated and may play a crucial role during HSC development. As the activity of RiBi mainly affects protein translation efficacy, we also performed protein synthesis assay (O-Propargyl-puromycin, OP-puro) to measure the physiological translation rates in E10.5 caudal half AECs (CD41^−^CD43^−^CD45^−^CD31^+^CD201^−^Kit^−^CD44^+^), HECs (PK44) and E11.0 AGM T1 pre-HSCs (CD31^+^CD45^−^CD41^low^). Notably, higher translation rates were observed in HECs compared with AECs and with T1 pre-HSCs (Fig. S3I-L), showing both an increased proportion of OP-puro^+/high^ cells (Fig. S3J) and an enhanced mean fluorescence intensity (MFI) of OP-puro signal in HECs (Fig. S3K,L), which is consistent with the higher RiBi activity in HECs.

### Disruption of RiBi impairs the generation of HSCs

**Fig. 2.**
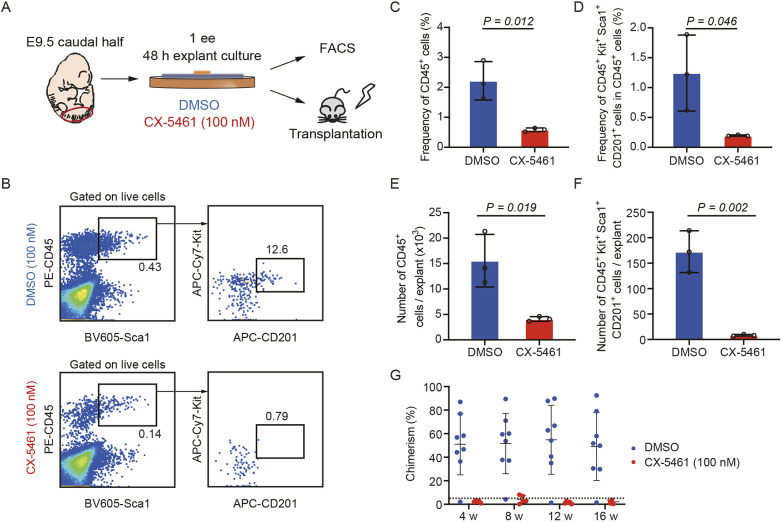
**Inhibiting RiBi impairs the generation of HSPCs.** (A) Schematic of explant cultures of E9.5 (27-29 somite pairs) caudal half regions with the treatment of DMSO and CX-5461. (B) Representative flow cytometric analysis of the progenies of E9.5 (27-29 somite pairs) caudal half explant culture. (C,D) Quantification of the frequency of hematopoietic cells (C; CD45^+^) and HSPCs (D; CD45^+^Kit^+^Sca1^+^CD201^+^) generated from the derivatives of the explant culture. Data are collected from three independent experiments. Data are mean±s.d. and are analyzed using an unpaired two-tailed Student's *t*-test. (E,F) Quantification of the number of hematopoietic cells (E; CD45^+^) and HSPCs (F; CD45^+^Kit^+^Sca1^+^CD201^+^) generated from the derivatives of the explant culture. Data are collected from three independent experiments. Data are mean±s.d. and were analyzed using an unpaired two-tailed Student's *t*-test. (G) Donor chimerism in the peripheral blood of recipients after direct transplantation of the derivatives of the explant culture (1 embryo equivalent per recipient). Data are mean±s.e.m.

To determine the role of RiBi in HSC generation, we sorted CD31^+^CD45^−^ cells from E10.0 AGM, which contains the majority of hematopoietic precursors. Subsequently, these cells were treated with CX-5461, a specific inhibitor of RNA polymerase I that has previously been reported to disturb rRNA transcription and, ultimately, RiBi (Cai et al., 2015; Prakash et al., 2019). CX-5461 treatment could effectively restrain the expression level of 47S pre-rRNA in CD31^+^CD45^−^ cells from E9.5 caudal half regions (Fig. S2C). Of note, after the co-culture assay, the proportion of hematopoietic cells (CD45^+^Kit^+^) exhibited a significant decrease that was concomitant with increasing concentrations of CX-5461 (Fig. S2A,B). This observation was further substantiated by an explant culture assay, which demonstrated a notable decline in both the number and proportion of CD45^+^ hematopoietic cells, as well as in the number and proportion of immunophenotypic hematopoietic stem and progenitor cells (HSPCs, CD45^+^Kit^+^Sca1^+^CD201^+^) after treatment with CX5461 (Fig. 2A-F). Importantly, the transplantation experiments using the derivatives of explant cultures showed significantly lower chimerism in the peripheral blood at 16 weeks post-transplantation in CX5461 treatment groups when compared with controls (Fig. 2G), suggesting that blocking RiBi dramatically hindered the production of adult repopulating HSCs. Taken together, these results validated that RiBi was pivotal for the generation of HSCs during embryonic development.

### Interfering RiBi prevents the generation of HSCs from HECs rather than pre-HSCs

**Fig. 3.**
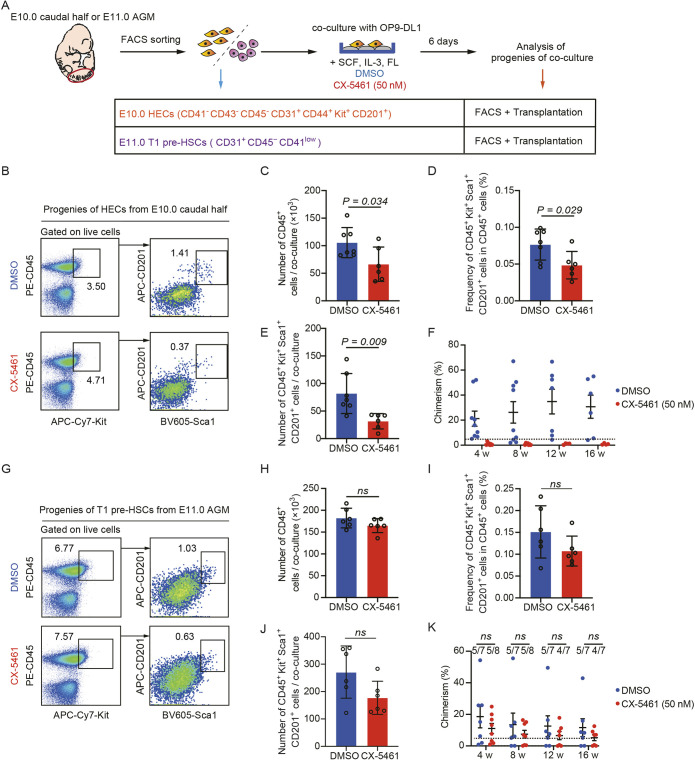
**RiBi deficiency selectively affects the function of HECs.** (A) Schematic illustration of hematopoietic induction of E10.0 caudal half HECs (32-34 somite pairs, CD41^−^CD43^−^CD45^−^CD31^+^CD201^+^Kit^+^CD44^+^) or E11.0 AGM T1 pre-HSCs (41-43 somite pairs, CD31^+^CD45^−^CD41^low^). (B) Representative flow cytometric analysis of the progenies of E10.0 caudal half HEC (CD41^−^CD43^−^CD45^−^CD31^+^CD201^+^Kit^+^CD44^+^) co-culture (1 ee per experiment). (C) Quantification of the number of hematopoietic cells (CD45+) generated from E10.0 caudal half HEC (CD41^−^CD43^−^CD45^−^CD31^+^CD201^+^Kit^+^CD44^+^) co-culture, treated with DMSO (*n*=7) or CX-5461 (*n*=6). Data are mean±s.d. and were analyzed using an unpaired two-tailed Student's *t*-test. (D,E) Quantification of the frequency and number of HSPCs (CD45^+^ Kit^+^ Sca1^+^ CD201^+^) generated from E10.0 caudal half HEC (CD41^−^CD43^−^CD45^−^CD31^+^CD201^+^Kit^+^CD44^+^) co-culture, treated with DMSO (*n*=7) or CX-5461 (*n*=6). Data are mean±s.d. and were analyzed using an unpaired two-tailed Student's *t*-test. (F) Peripheral blood donor chimerism in recipients after direct transplantation of derivatives from E10.0 caudal half HEC (CD41^−^CD43^−^CD45^−^CD31^+^CD201^+^Kit^+^CD44^+^) co-culture (4 ee per recipient). Data are mean±s.e.m. (G) Representative flow cytometric analysis of progenies from E11.0 AGM T1 pre-HSC (CD31^+^CD45^−^CD41^low^) co-culture (1 ee per experiment). (H) Quantification of the number of hematopoietic cells (CD45^+^) generated from E11.0 AGM T1 pre-HSC (CD31^+^CD45^−^CD41^low^) co-culture, treated with DMSO (*n*=6) or CX-5461 (*n*=6). Data are mean±s.d. and were analyzed using an unpaired two-tailed Student's *t*-test. (I,J) Quantification of the frequency and number of HSPCs (CD45^+^Kit^+^Sca1^+^CD201^+^) generated from E11.0 AGM T1 pre-HSC (CD31^+^CD45^−^CD41^low^) co-culture, treated with DMSO (*n*=6) or CX-5461 (*n*=6). Data are mean±s.d. and were analyzed using an unpaired two-tailed Student's *t*-test. (K) Peripheral blood donor chimerism in recipients after direct transplantation of derivatives from E11.0 AGM T1 pre-HSC (CD31^+^CD45^−^CD41^low^) co-culture (1 ee per recipient). Data are mean±s.e.m.

The generation of HSCs is a complex and tightly regulated process that primarily involves initial HEC specification, followed by a fate transition to pre-HSCs, which ultimately acquire definitive hematopoietic function. We then separately sorted HECs (PK44) from the E10.0 caudal half and T1 pre-HSCs (CD31^+^CD45^−^CD41^low^) from the E11.0 AGM, as previously identified (Zhou et al., 2016; Hou et al., 2020), and combined flow cytometric analysis and transplantation assays to determine the stages at which the defect occurred due to the inhibition of RiBi (Fig. 3A; Table S1). After the co-culture of HECs, the number of CD45^+^ hematopoietic cells was significantly reduced in groups treated with CX-5461 compared with the controls (Fig. 3B). In particular, both the quantity and proportion of the immunophenotypic HSPCs (CD45^+^Kit^+^Sca1^+^CD201^+^) also substantially diminished upon treatment with CX-5461 (Fig. 3C-E). This finding was further validated by transplantation experiments, which demonstrated a failure in reconstitution in CX-5461 treatment groups at 16 weeks post-transplantation, in contrast to the controls (Fig. 3F). PK44-represented HECs exhibit a continuum of cellular states of the EHT, encompassing the endothelial, dual endothelial and hematopoietic, and hematopoietic potential (Hou et al., 2020; Wang et al., 2023). We further found CX-5461 treatment only led to a loss in the hemogenic capacity of PK44 cells, while leaving endothelial potential unaffected (Fig. S3A-C). Nevertheless, upon conducting co-culture experiments with T1 pre-HSCs (CD31^+^CD41^low^CD45^−^), we observed that, although the progenies of T1 pre-HSCs presented a slight reduction in CX-5461 treatment groups, inhibition of RiBi in T1 pre-HSCs did not compromise their ability to generate functional HSCs (Fig. 3G-K). We also used two additional specific RNA polymerase I inhibitors, CX-3543 and BMH-21 (Baweja and Subudhi, 2021), to further verify our findings. Similarly, CX-3543 and BMH-21 treatment both effectively restrain the expression level of 47S pre-rRNA in sorted CD31^+^CD45^−^ cells in E10.0 caudal half regions (Fig. S2D). Notably, after the co-culture of HECs, the proportion of immunophenotypic HSPCs (CD45^+^Kit^+^Sca1^+^CD201^+^) was dramatically reduced in response to CX-3543 and BMH-21 treatment when compared with the controls (Fig. S2E,G). Moreover, there was still no significant difference observed upon conducting co-culture experiments with T1 pre-HSCs (Fig. S2F,H). Overall, these results suggest that RiBi acts at the HEC stage but is not required for subsequent pre-HSC maturation to form functional HSCs.

### Perturbation of RiBi in HECs specifically delays their cell cycle progresses

**Fig. 4.**
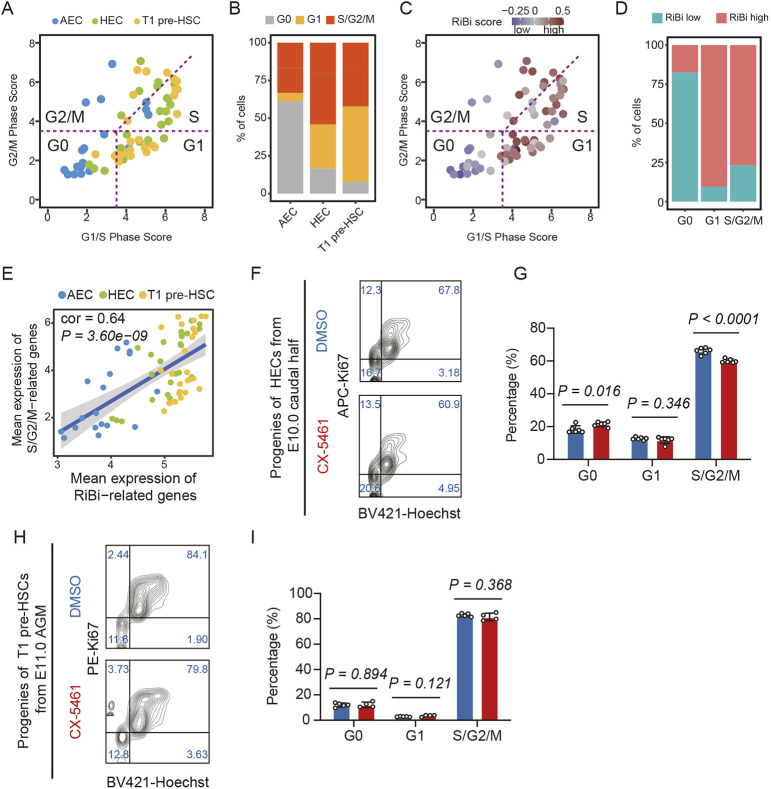
**RiBi deficiency leads to cell cycle arrest in HECs.** (A) Categorizing cell cycle phases by evaluating the average expression of G1/S and G2/M gene sets. (B) Stacked bar chart displaying the composition of different cell cycle phases within the three respective cell clusters. (C) Dot plot illustrating that the RiBi scores corresponded to cell cycle status. A RiBi score greater than 0 is classified as RiBi high, whereas a score less than 0 is categorized as RiBi low. (D) Stacked bar chart depicting the distribution of activity of RiBi levels in different cell cycle phases. (E) Correlation between S/G2/M and RiBi-related genes. The colors of the points indicate the clusters, while the linear fit is depicted as a blue line, with the confidence interval represented by a gray area. The Pearson correlation coefficient is used to assess the correlation between the two. An absolute value of correlation coefficient greater than 0.5 and a *P* value of less than 0.05 is considered indicative of a strong correlation. (F) Representative flow cytometric analysis illustrating the cell cycle status of progenies derived from E10.0 caudal half HECs (CD41^−^CD43^−^CD45^−^CD31^+^CD201^+^Kit^+^CD44^+^) after a 48 h co-culture. (G) Bar plot showing the percentage of cells in G0, G1 and S/G2/M phase from F. Data are collected from four independent experiments. Data are mean±s.d. and were analyzed using an unpaired two-tailed Student's *t*-test. (H) Representative flow cytometric analysis illustrating the cell cycle status of progenies derived from E11.0 AGM T1 pre-HSCs (CD31^+^CD45^−^CD41^low^) following a 48 h co-culture. (I) Bar plot showing the percentage of cells in G0, G1 and S/G2/M phase from H. Data are collected from four independent experiments. Data are mean±s.d. and were analyzed using an unpaired two-tailed Student's *t*-test.

A previous study indicated that the activation of cell cycle is essential for EHT and the ensuing HSPC generation (Canu et al., 2020). When compared with AECs and T1 pre-HSCs, HECs showed a more active cycling status, characterized by a greater proportion of cells in the S/G2/M phase (Fig. 4A,B; Table S1). Meanwhile, cells with higher RiBi level were more likely to enter the G1/S phase, and the expression of genes related to RiBi exhibited a positive correlation with those associated with the S/G2/M phases (Fig. 4C-E). As several studies have clarified that RiBi might be involved in regulating cell cycle progress (Prakash et al., 2019; Turi et al., 2019), we therefore hypothesized that inhibition of RiBi in HECs could affect the cell cycle, subsequently disrupting their hemogenic capacity. Of note, when HECs (PK44) were treated with CX-5461 for 48 h, there was a marked increase in the proportion of cells in the G0 phase and a corresponding decrease in cells in the S/G2/M phase, indicating the occurrence of delayed cell cycle progresses in HECs upon RiBi inhibition (Fig. 4F,G). To exclude the effect of CX-5461 on cell apoptosis or cell death, additional experiments were conducted to assess the cell death status, which revealed that there was no significant difference in derivatives of CD31^+^CD45^−^ cells for the two groups (Fig. S3D-G). Intriguingly, we also found that the distribution of each cell cycle phase in the progenies of T1 pre-HSCs (CD31^+^CD41^low^CD45^−^) remained unchanged after treatment with CX-5461 (Fig. 4H,I). In summary, these findings implied that RiBi might preserve the ability of HECs to generate HSCs though modulating progression of the cell cycle.

### Runx1 pre-configures the RiBi signature in AECs before HECs

**Fig. 5.**
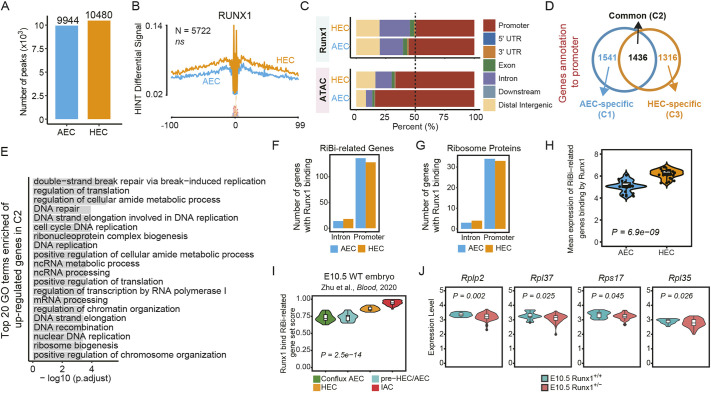
**Runx1 binds RiBi-related gene loci and mediates their expression.** (A) Quantification of the number of Runx1-binding peaks called by ChIP-seq data. (B) RUNX1 footprint in open chromatin of AECs and HECs using ATAC-seq data. The graphs present the average normalized read counts in conjunction with the *P*-values derived from the HINT differential analysis. *N* represents the count of nonredundant RUNX1 footprints, while ‘ns’ indicates that the results are not statistically significant (*P*>0.05). (C) Bar plots representing the genome annotation of peaks identified through the use of Runx1 ChIP-seq data and ATAC-seq data. (D) Venn diagram illustrating the number of genes annotated by Runx1-binding peaks located at promoters (2.5 kb upstream and downstream of the TSS) in AECs and HECs. (E) Top 20 enriched Gene Ontology biological process (GO: BP) terms by using genes upregulated in C2. (F,G) Quantification of the number of RiBi-related genes (F) and ribosome protein genes (G) with Runx1 binding at the promoter or intron regions. (H) Violin plot displaying the average expression of RiBi-related genes with Runx1 binding in AECs and HECs. (I) Violin plot showing the gene set score of four populations (Conflux AECs, pre-HECs, HECs and IACs) in a wild-type embryo, calculated using RiBi-related genes with Runx1 binding. (J) Violin plot displaying the average expression of ribosome proteins in pre-HECs for E10.5 *Runx1*^+/+^ and E10.5 *Runx1*^+/−^ littermates, calculated using RiBi-related genes with Runx1 binding. In H-J, a Wilcoxon rank sum test is used to evaluate the significance of difference; the *P* values are provided to indicate the statistical significance of the comparison. *P*<0.05 is considered statistically significant.

A previous study has elucidated that Runx1 appears to directly regulate RiBi in mouse adult bone marrow HSPCs (Cai et al., 2015). To explore whether RiBi is also regulated by Runx1 during EHT, we employed and re-analyzed Runx1 ChIP-seq data from AECs to HECs in our previous report (Li et al., 2022; GEO accession number GSE161328). First, the number of Runx1-binding peaks in the genome are similar between AECs and HECs (Fig. 5A,C; Fig. S4A; Table S1). Consistent with this was the observation that the amount of open genomic regions tending to be occupied by Runx1 was also similar between AECs and HECs, as evidenced by ATAC-seq data (Fig. 5B,C). Second, we observed that over half of the Runx1-binding peaks were annotated to promoter regions and exhibited three major enrichment patterns, comprising AEC-specific (C1), HEC-specific (C3) and common shared in AECs and HECs (C2) (Fig. 5C,D). Furthermore, we conducted Gene Ontology (GO) analysis on the genes that exhibited Runx1 binding within their promoter regions accompanied with upregulated expression. We found enrichment of several RiBi-related terms as early as those in C1, including ribonucleoprotein complex export from the nucleus and ribonucleoprotein complex localization. More specific terms, such as ribonucleoprotein complex biogenesis, regulation of transcription by RNA polymerase I and ribosome biogenesis, were significantly enriched in C2 (Fig. 5E, Fig. S4B-D). We also found that Runx1 exhibited a preference for binding to promoters rather than to introns of RiBi-related genes, as well as to the genes encoding ribosomal proteins in both AECs and HECs. The expression level of these genes was upregulated in HECs compared with AECs (Fig. 5F-H; Table S2). Collectively, these results suggested that Runx1 already binds to RiBi-related genes in AECs to facilitate the subsequent upregulation of these genes in HECs.

Next, we used and carried out a deep-analysis of the published scRNA-seq data (Zhu et al., 2020) of EHT-related cells from *Runx1^+/+^* and *Runx1^+/−^* littermates, in which the identified pre-HEC transcriptomically corresponds to the AEC we annotated (Hou et al., 2020; Wang et al., 2022). We indeed observed a continuous increase in RiBi levels along the developmental path from conflux AECs to IACs in *Runx1^+/+^* (wild-type) embryos (Fig. 5I). Notably, Runx1 haploinsufficiency resulted in an increase in the proportion of pre-HECs/AECs compared with control (Zhu et al., 2020). Strikingly, the expression levels of several ribosomal protein genes in pre-HECs/AECs were indeed found to be reduced in *Runx1*^+/−^ compared with in *Runx1*^+/+^ (Fig. 5J), further confirming the notion that Runx1 was involved in modulating RiBi gene expression as early as in AECs. In summary, these results together demonstrate that the binding of Runx1 to the genome participating in regulating RiBi gene expression might contribute to HSC development.

## DISCUSSION

Excavating molecular regulatory mechanisms of HSC ontogeny can provide new clues for HSC regeneration *in vitro*. Many studies have shown that EHT is precisely regulated by several master transcription factors and signaling pathways (Adamo et al., 2009; Chen et al., 2009; Gering and Patient, 2010; Fitch et al., 2012; Marcelo et al., 2013; Espín-Palazón et al., 2014; Lee et al., 2014; Li et al., 2014; Sawamiphak et al., 2014; Butko et al., 2015; Diaz et al., 2015; Gama-Norton et al., 2015; Monteiro et al., 2016; Dzierzak and Bigas, 2018; Kang et al., 2018; Yang et al., 2022; Coulombe et al., 2023). Some fundamental biological processes, such as metabolism and cell cycle, have also been reported to be involved in the regulation of HSC development (Harris et al., 2013; Canu et al., 2020; Oburoglu et al., 2022). Recent studies indicated the pivotal role of RiBi in orchestrating the homeostasis and fate decisions of stem cells (Sanchez et al., 2016; Khajuria et al., 2018; Lv et al., 2021; Yu et al., 2021). However, the precise significance of RiBi in HSC development remains inadequately elucidated.

Preliminarily, we found that robust RiBi was markedly augmented from the HEC stage along the HSC ontogeny. Further screening revealed that the synthesis of rRNA, the initial stage of RiBi, as well as muti-steps of RiBi, were significantly enhanced in HECs. Currently, selectively inhibiting RiBi within a specific cell population *in vivo* is exceedingly challenging. The selective molecule CX-5461 was therefore identified as an effective RiBi inhibitor and used to study the function of RiBi in HSC generation (Drygin et al., 2011; Prakash et al., 2019; Kang et al., 2021). Functionally, through organ culture and transplantation experiments, we observed that reduced RiBi blocked the generation of HSCs in AGM region. Subsequently, *in vitro* and *ex vivo* experiments clarified that decreased RiBi hampers the generation of HSCs from HECs, rather than T1 pre-HSCs. Mechanistically, we discovered that reduced RiBi in HECs leads to more cells stagnating in G0 phase. It has been documented that blocking the cell cycle in HECs would interrupt the generation of HSPCs (Canu et al., 2020).

However, how RiBi regulated the cell cycle progression in HECs still needs to be explored. Shi et al. discovered that core ribosomal proteins exhibit heterogeneity in constituting translating ribosomes, which seems to facilitate the preferential translation of certain mRNAs (Shi et al., 2017). In line with this observation, our findings indicate that numerous ribosomal proteins and associated factors display distinct stage-specific expression patterns (Fig. S1B). This implies the existence of specialized translation events that could be substantiated by single-cell ribosome profiling (scRibo-seq) in future studies, as the technology becomes more widespread. Moreover, through the integration analysis of omics data, we uncovered that Runx1 bound to RiBi-related gene loci and upregulated their expression in HECs compared with AECs. Previous studies have shown that Runx1 haploinsufficiency interrupted the transition of pre-HECs/AECs to HECs and subsequent HSC generation (Zhu et al., 2020). Combined with our findings in this study, we preliminarily concluded that RiBi is a downstream target of Runx1, and the effect of inhibiting RiBi is similar to that of Runx1 deletion during HSC development.

In summary, our work sheds light on the essential role of RiBi in HSC development and preliminarily expounds its possible upstream and downstream regulatory mechanisms. These findings enhance the comprehension of the precise regulatory mechanisms underlying EHT and offer a previously unreported perspective for the *in vitro* regeneration of HSCs.

## MATERIALS AND METHODS

### Mice

All mice were maintained on C57BL/6 genetic background and bred in specific pathogen free (SPF) conditions at the Laboratory Animal Center of Academy of Military Medical Sciences. E9.5-E11.5 embryos were confirmed by counting the somite pairs (sp). The caudal half or AGM region was dissected as previously reported (Rybtsov et al., 2011). The experimental manipulations of mice were approved by the Animal Care and Use Committee of the Academy of Military Medical Sciences.

### Explant culture

Caudal half regions were isolated from E9.5 embryos and cultured using an *ex vivo* explant culture system, as previously described (Medvinsky and Dzierzak, 1996). The medium used for the caudal half region culture contained IMDM (Hyclone) and 20% fetal bovine serum (Hyclone) in the presence or absence of 100 nM CX-5461 (MCE, HY-13323). After being cultured for 48 h, caudal half regions were dissociated in collagenase for flow cytometry analysis and transplantation.

### OP9-DL1 co-culture

The OP9-DL1 and OP9 stromal cell line were kindly provided by Y. Zhang (Peking University, Beijing, China). FACS-sorted cells were co-cultured with OP9-DL1 stromal cells in 24-well plates containing α-MEM (Gibco), 10% fetal bovine serum (Hyclone) and cytokines (100 ng/ml SCF, 100 ng/ml Flt3 ligand and 100 ng/ml IL-3, PeproTech) in the presence or absence of 50 nM CX-5461 (MCE, HY-13323), 50 nM CX-3543 (MCE, HY-14776) and 20 nM BMH-21 (MCE, HY-12484). After 6 days of co-culture, cells in each well were harvested for flow cytometry analysis and transplantation.

### Transplantation assay

For transplantation of freshly isolated cells from explant culture or co-cultured cells from HECs, 10- to 12-week-old female recipients (CD45.2/2) were subjected to a split dose of 9 Gy γ-irradiation (^60^Co) at an interval of 2 h. Donor cells (CD45.1/1), together with 2×10^4^ nucleated bone marrow cells (CD45.2/2), were injected into irradiated adult recipients (CD45.2/2) via the tail vein. For transplantation of co-cultured cells from T1 pre-HSCs, 10- to 12-week-old female recipients (CD45.1/1) were subjected to a split dose of 9 Gy γ-irradiation (^60^Co) at an interval of 2 h. Donor cells (CD45.2/2), together with 2×10^4^ nucleated bone marrow cells (CD45.1/1), were injected into irradiated adult recipients (CD45.1/1) via the tail vein. The recipients demonstrating at least 5% donor-derived chimerism in peripheral blood were counted as successfully reconstituted.

### Cell cycle detection

For cell cycle analysis by Hoechst/Ki67 staining, cells were fixed using Fixation and Permeabilization Solution (BD Biosciences, 554722). After surface marker antibody staining, anti-Ki67 (eBioscience, SolA15) and Hoechst 33342 (BD, 561908) staining were performed following a standard protocol (Kim and Sederstrom, 2015).

### Flow cytometry

Cells were analyzed and sorted using the flow cytometer Calibur or Aria 2 (BD Biosciences). Data were analyzed with FlowJo software (Tree Star). The following antibodies were used for staining of the cells: CD31 (MEC13.3), CD41 (MWReg30), CD45 (30-F11), c-Kit (2B8), CD201 (eBio 1560), CD45.1 (A20), CD45.2 (104), Ter119 (TER-119), VE-cadherin (eBioBV13), Sca-1 (D7), Ki67 (SolA15), 7-amino-actinomycin D (7-AAD) and Streptavidin APC-eFluor 780. All monoclonal antibodies and 7-AAD were purchased from eBioscience, except for CD31 and CD41 (from BD Pharmingen), and CD150 and VE-cadherin (from BioLegend). Antibodies were diluted in 2% FBS/PBS and used at 1:500. For single-cell sorting, rainbow beads were used to confirm that exactly one single cell was sorted into each well.

### qPCR

After sorting 100 cells straight into the cell lysis buffer, the previously described procedures for reverse transcription and cDNA amplification were carried out (Gao et al., 2018). qRT–PCR was performed on a LightCycler 480 II system (Roche Diagnostics). The sequences of primers were: 47S pre-rRNA (5′ETS amplicons), ACACGCTGTCCTTTCCCTATTA (sense) and CCCAAGCCAGTAAAAAGAATAGG (anti-sense); β-actin, GTAAAGACCTCTATGCCAACAC (sense) and ATGATCTTGATCTTCATGGTGCTA (anti-sense).

### OP9-DL1-based hematopoietic and endothelial potential assay

FACS-sorted cells were plated on the OP9-DL1 stromal cells in IMDM (Hyclone) containing 15% fetal bovine serum (Hyclone), 1% bovine serum albumin (Sigma), 10 μg/ml insulin (Macgene), 200 μg/ml transferrin (Sigma), 5.5×10^−5^ mol/l 2-mercaptoethanol (Gibco) and cytokines (100 ng/ml rhVEGF-165 and 50 ng/ml SCF, PeproTech) in the presence or absence of 50 nM CX-5461 (MCE, HY-13323) (Hou et al., 2020). After 6 days of co-culture, cells were fixed for 30 min in 2% paraformaldehyde and stained with CD45 antibody (BD Biosciences) to assess the generation of hematopoietic progeny. CD31 (BD Pharmingen, MEC13.3) immunohistochemical staining was carried out following conventional protocols (Hou et al., 2020), and the formation of CD31-positive tubules in the wells was regarded as indicative of endothelial potential.

### Protein synthesis assay (O-propargyl-puromycin, OP-puro)

The Protein Synthesis Assay Kit (Abcam, ab239725) was used to measure translation rates in AECs, HECs and pre-HSCs. Briefly, E10.5 caudal half and E11.0 AGM were dissected into single cell suspension by type I collagenase digestion. After neutralizing and resuspended with culture medium, 400× O-propargyl-puromycin was added to the cells, and incubated for 1 h in a 37°C incubator. Labeled cells were then fixed and treated following standard procedures. After that, cells were labeled as surface antibody cocktails and analyzed by FACS.

### Curated gene sets

Several gene sets were curated from the published data. The set of genes associated with RiBi was curated from the Gene Ontology (GO) database (http://geneontology.org/) (Ashburner et al., 2000). To accomplish this, genes involved in biological processes were meticulously searched using the keywords ‘mouse’, ‘ribosome’ and ‘rRNA’. Ultimately, 529 genes were obtained as RiBi-related genes for subsequent analysis. The genes, including *Gja4*, *Unc5b*, *Mecom*, *Hey1*, *Efnb2*, *Dll4*, *Vegfc*, *Epas1*, *Cxcr4* and *Igfbp3*, were characterized as arterial endothelia features (Hou et al., 2020). A comprehensive set of 98 genes has been designated to depict the features of hematopoietic cells (Zhou et al., 2016).

### Processing and integration of scRNA-seq data

Cellranger was employed for the trimming, alignment and quantitation of 10x scRNA-seq data. The following outlines the quantification process for STRT scRNA-seq analysis. Herein, the UMI-based scRNA-seq approach was used to quantify the gene expression profile within individual cells. Initially, Cutadapt (v4.4) was applied to trim Read 1 to remove the TSO sequence and polyA tail segment. Reads contaminated with adapter sequences or plagued by low-quality bases (*n*>10%) are excluded. Subsequently, TopHat v2.0.12 (Trapnell et al., 2009) was used to perform the alignment against the mm10 mouse transcriptome database (UCSC). Unique molecular identifiers (UMIs) coupled with gene pairs were quantified using HTSeq (Anders et al., 2015), with subsequent categorization according to cell-specific barcodes. Culminating the analysis, the abundance of unique UMIs attributed to each gene within solitary cells was equated to the corresponding transcript copy number.

### Principal component analysis and dimensionality reduction

The Seurat R package (v4.9) (Hao et al., 2021) was employed to analyze the scRNA-seq data, facilitating the identification of highly variable genes (HVGs) and differentially expressed genes (DEGs), as well as dimensionality reduction via PCA or t-SNE, unsupervised clustering, among other analyses. The standard analytical workflow was succinctly outlined here. First, genes expressed in at least three individual cells were retained. Subsequently, the ‘FindVariableGenes’ function was engaged to pinpoint genes with significant variability based on log-transformed expression values, specifically log2(TPM/10+1). Genes manifesting an average expression value exceeding 1 but below 8, and a dispersion metric greater than 1, were recognized as highly variable genes (HVGs). Furthermore, to investigate whether RiBi can serve as a discriminative biomarker for the various developmental stages of HSC, we directly used a collection of 529 RiBi-related genes, as well as a set of 529 genes selected at random, as HVGs for the ensuing principal component analysis (PCA). Subsequently, the Elbow method was employed to determine the most significant principal components (PCs) that are informative for the forthcoming dimensionality reduction using t-SNE and for the subsequent graph-based clustering analysis (Levine et al., 2015).

### Identification of signature genes and GO enrichment

The ‘FindMarkers’ or ‘FindAllMarkers’ function, using the default Wilcoxon rank sum test, was deployed to identify signature genes. For GO enrichment analysis, Metascape (http://metascape.org) (Zhou et al., 2019) and clusterProfiler (Wu et al., 2021) were used. Within these analyses, biological processes with *P*-values corrected by the Benjamini-Hochberg method below 0.05 are defined as significantly enriched categories within the respective population.

### Processing of ChIP-seq/ATAC-seq data

The processing steps of ChIP-seq/ATAC-seq data are as follows. First, FastQC (v.0.11.8) was used to assess the quality of the sequencing data. Subsequently, fastp software (Chen et al., 2018) was employed to trim low-quality reads and adapter sequences. Then, bowtie2 (Langdon, 2015) was used to align the processed read segments to the mm10 reference genome, setting the maximum number of allowed mismatches to 1. Only alignments with a mapping quality score exceeding 30 were considered uniquely mapped reads. Next, the aligned reads were sorted using Samtools (Li et al., 2009), and the sorted files were used as input for Picard software (v.2.2.4), where the MarkDuplicates function was applied to remove duplicate reads for downstream analysis. To account for potential sequencing depth biases across different batches, Samtools (Li et al., 2009) was again used to randomly down sample the reads and standardize them to a uniform level. Finally, the bamCoverage function in deepTools (v1.5.12) (Ramírez et al., 2016) was used to generate bigWig files, which facilitate subsequent visualization in the Integrative Genomics Viewer (IGV) or on the UCSC Genome Browser (https://genome.ucsc.edu/). MACS2 software (Zhang et al., 2008; Liu, 2014) (v2.2.6) was employed to identify peaks within ChIP-seq/ATAC-seq data. Here, the enrichment mode selected is ‘—narrow’. Enrichment peak annotations across the entire genome were carried out using the ChIPseeker (v1.20.0) (Yu et al., 2015) and clusterProfiler (Wu et al., 2021). Specifically, the promoter region has been defined as the area spanning 3 kb both upstream and downstream of the transcription start site (TSS).

### GSVA analysis

Gene set variation analysis (GSVA) (Hänzelmann et al., 2013) facilitates the transformation of gene-level expression profiles pertaining to gene sets associated with KEGG pathways (Kanehisa et al., 2017) into an enrichment score profile of corresponding biological processes. The ssGSEA (Barbie et al., 2009) method was employed to estimate the gene set enrichment score for each cell. A two-sample Wilcoxon test was used to assess differential enrichment across two populations, with corrected *P*-values below 0.05 deemed statistically significant.

### Correlation analysis

The average expression of the focused gene set was calculated for each cell. The relevant developmental cell populations of interest were then selected for computing the Pearson correlation coefficient.

### Cell cycle analysis

The method similar to that of Tirosh et al. (2016b) was employed for categorizing the cell cycle stages. Specifically, the average gene expression for the 43 G1/S (Macosko et al., 2015) and 54 G2/M (Tirosh et al., 2016a) genes was calculated in each cell, which was used to determine G1/S and G2/M scores. These scores were then used to approximate the cell cycle stage based on their distribution. Cells with both G1/S and G2/M scores below 3.5 were categorized as ‘G0’ phase, whereas scores exceeding this threshold indicated a proliferative condition. Within the subset of proliferative cells, those with G2/M scores surpassing their G1/S equivalents were classified as being in the ‘G2/M’ phase. Conversely, if the G1/S score trumped that of G2/M and the latter was below 3.5, the cells were assigned to the ‘G1’ phase. Should the G2/M score meet or exceed 3.5, the cell was subsequently labeled as being in the ‘S’ phase.

### Footprint analysis

The HINT (Li et al., 2019) software was used for ATAC-seq footprint analysis, leveraging a Hidden Markov Model (HMM) framework. Concisely, footprints were delineated within regions of chromatin accessibility, identified as peaks by MACS2 v2.2.6 (Zhang et al., 2008; Liu, 2014) within merged replicates for each sample. These footprints were subsequently aligned with TF motifs from the JASPAR database. HINT-differential was used to evaluate the differential activity for each TF between two cell populations.

### Statistical analysis

No statistical methods were used to predetermine sample size. For statistical analysis between groups, an unpaired two-tailed Student's *t*-test was used to calculate *P*-values, unless otherwise specified. Statistical analysis was carried out using GraphPad Prism 8.

## Supplementary Material

10.1242/develop.202875_sup1 Supplementary information

Table S1. Immunophenotype, location and developmental stages of cell populations.

Table S2. Genes with RUNX1 binding in AECs and HECs.
