# Supplementary material for: Ribosome biogenesis is essential for hemogenic endothelial cells to generate hematopoietic stem cells
Source: Development. 2024 Oct 23;151(21):dev202875. doi: 10.1242/dev.202875 (PMC11529273; doi:10.1242/dev.202875)
Supplement: Supplementary information [file develop-151-202875-s1.pdf]

Supplemental Figure 1

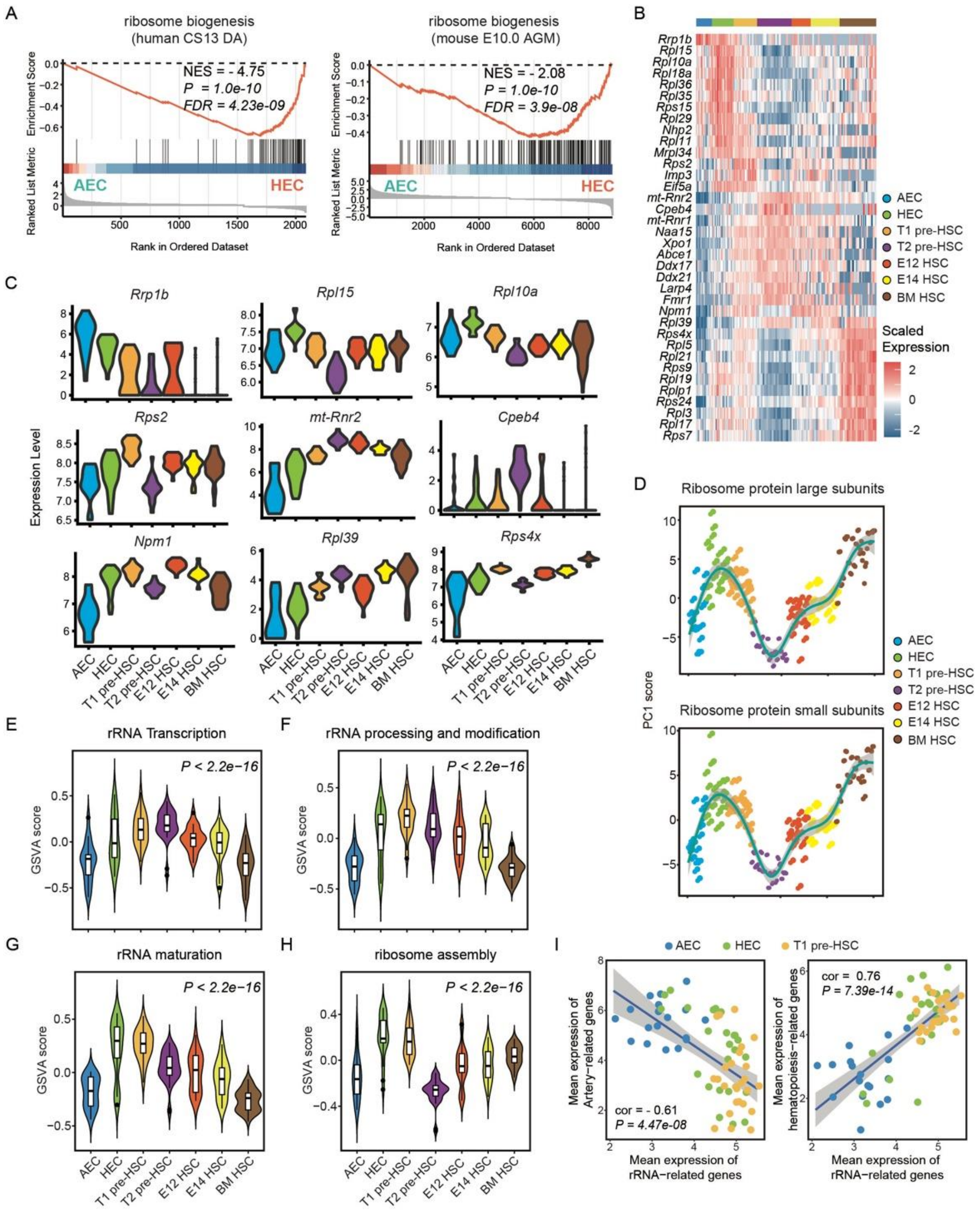

Fig. S1. The dynamic changes of RiBi during HSC development.

A. Gene Set Enrichment Analysis (GSEA) of ribosome biogenesis (GO:0042254) process between AECs and HECs in human (left) and mouse (right). DA, dorsal aorta; AGM, aorta-gonad-mesonephros. The nominal p-value and normalized enrichment score (NES) are provided to indicate the statistical significance and strength of the enrichment, respectively. Nominal P value, empirical phenotype-based permutation test ($P < 0.05$, $FDR < 0.25$).

Development • Supplementary information

B. Heatmap depicting relative expression of RiBi-related signature genes, distinguished by their significantly higher expression (adjusted P < 0.01, fold change > 1.5) within each cluster versus other clusters, across defined cell populations.

C. Violin plots showing the expression levels of indicated genes defined as $\log_2$(TPM+1).

D. Scatter plots displaying the change of ribosomal large/small subunits via PC1 scores, aligned with average expression for correct direction, and include loess-fit curves with 95% confidence intervals.

E-H. Violin plots illustrating the GSVA score of ‘rRNA transcription’ (E), ‘rRNA processing and modification’ (F), ‘rRNA maturation’ (G) and ‘ribosome assembly’ (H) during HSC development, with statistical significance assessed via the Kolmogorov-Smirnov test across seven cell clusters.

I. Correlation between artery/hematopoiesis signature genes and rRNA-related genes, color-coded by cell clusters, with a blue linear fit line and gray confidence interval shading. The Pearson correlation coefficient is utilized to assess the correlation between the two. Absolute value of correlation coefficient greater than 0.5 and P value less than 0.05 is considered indicative of a strong correlation.

Development • Supplementary information

Supplemental Figure 2

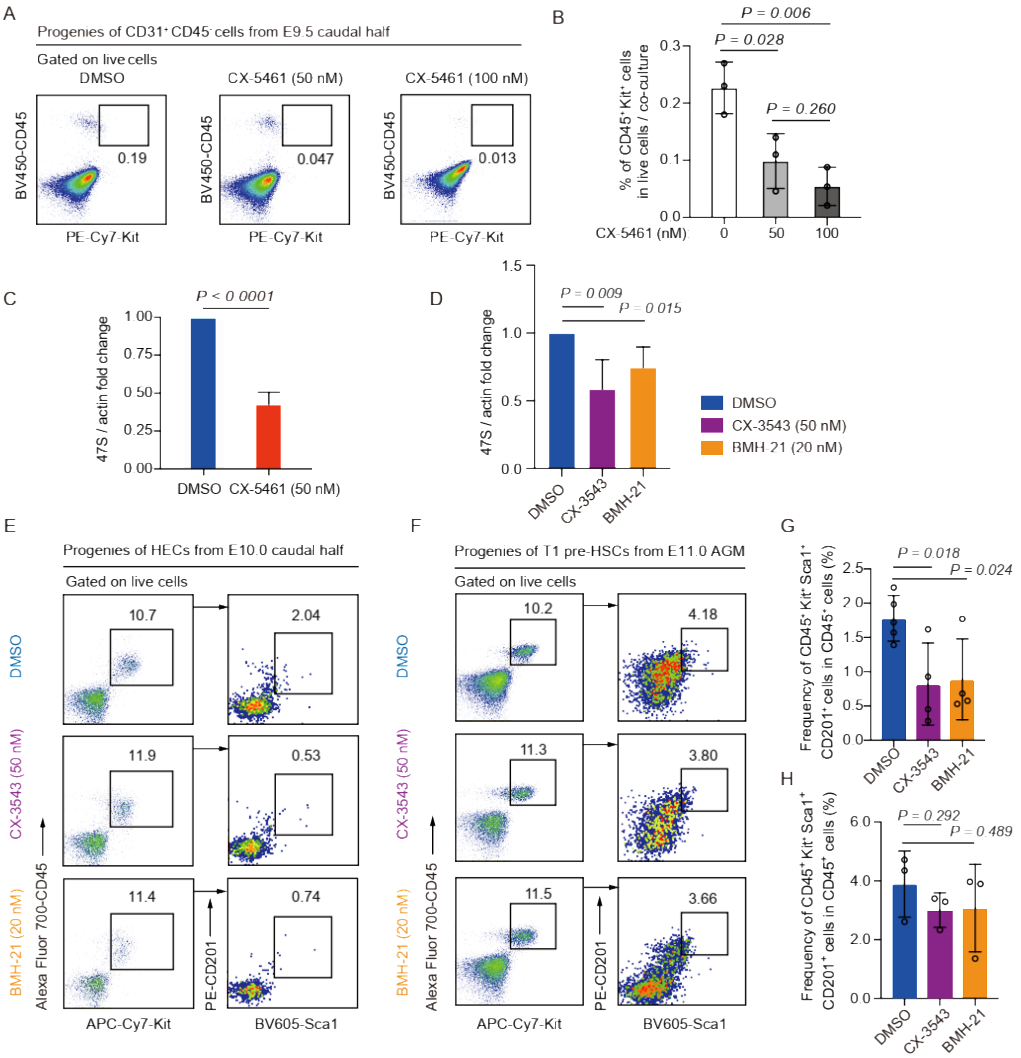

Fig. S2. CX-5461 is used to inhibit RiBi.

A. Representative flow cytometric analysis of the progenies of 100 $CD31^+CD45^-$ cells from E9.5 caudal half regions co-culture after treatment with increasing concentrations of CX-5461.

B. Quantification of the frequency of $CD45^+Kit^+$ cells in the derivatives of the co-culture from A. Data are collected from three independent experiments. Data are represented as mean ± SD and analyzed by unpaired two-tailed Student's t-test.

C. qRT-PCR detecting the expression level of 47S pre-rRNA in sorted $CD31^+CD45^-$ cells in E9.5 caudal half regions with the treatment of CX-5461. Data are collected from three independent experiments, and statistical differences were assessed using an unpaired two-tailed Student's t-test.

D. qRT-PCR detecting the expression level of 47S pre-rRNA in sorted $CD31^{+}CD45^{-}$ cells in E10.0 caudal half regions with the treatment of CX-3543 and BMH-21. Data are collected from three independent experiments, and statistical differences were assessed using an unpaired two-tailed Student’s t-test.

E. Representative flow cytometric analysis of the progenies of E10.0 caudal half HECs ($CD41^{-}CD43^{-}CD45^{-}CD31^{+}CD201^{+}Kit^{+}CD44^{+}$) co-culture.

F. Representative flow cytometric analysis of progenies from E11.0 AGM T1 pre-HSCs ($CD31^{+}CD45^{-}CD41^{low}$) co-culture.

G. Quantification of the frequency of HSPCs ($CD45^{+}Kit^{+}Sca1^{+}CD201^{+}$) generated from E10.0 caudal half HECs ($CD41^{-}CD43^{-}CD45^{-}CD31^{+}CD201^{+}Kit^{+}CD44^{+}$) co-culture, DMSO (n = 5), CX-3543 (n = 4) and BMH-21 (n = 4), respectively. Data are represented as mean ± SD and analyzed by unpaired two-tailed Student’s t-test.

H. Quantification of the frequency of HSPCs ($CD45^{+}Kit^{+}Sca1^{+}CD201^{+}$) generated from E11.0 AGM T1 pre-HSCs ($CD31^{+}CD45^{-}CD41^{low}$) co-culture, DMSO (n = 3), CX-3543 (n = 3) and BMH-21 (n = 3), respectively. Data are represented as mean ± SD and analyzed by unpaired two-tailed Student’s t-test.

Development • Supplementary information

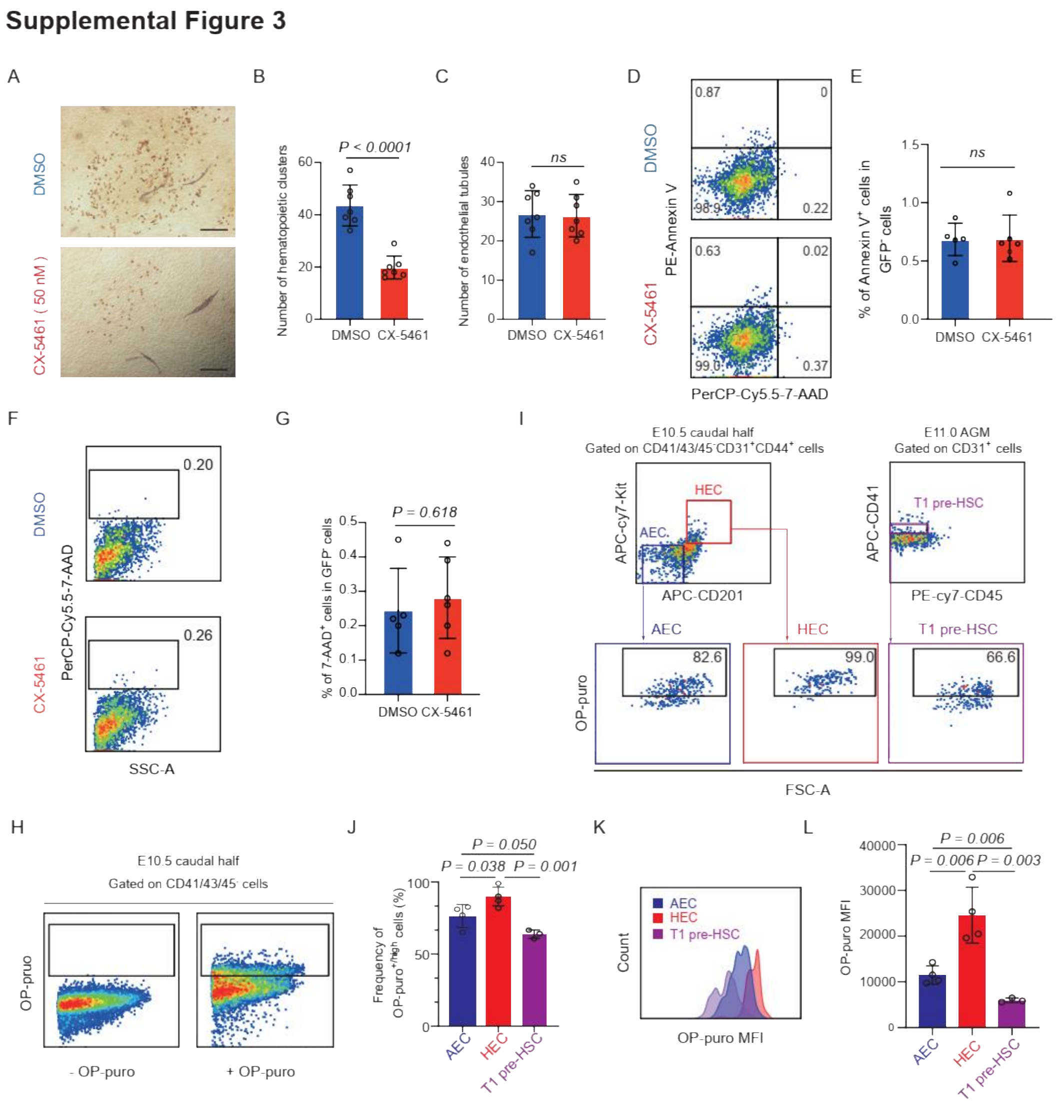

Fig. S3. RiBi deficiency selectively affects the function of HECs.

A. Representative CD31 and CD45 immunostaining on the cultures of 100 HECs ($CD41^-CD43^-CD45^-CD31^+CD201^+Kit^+CD44^+$) from E10.0 caudal half regions after treatment with CX-5461. Data are from three independent experiments. Scale bars, 100 μm.

B, C. Quantification of the number of hematopoietic clusters (B) and endothelial tubes (C). Data are represented as mean ± SD and analyzed by unpaired two-tailed Student’s t-test.

D. Representative flow cytometric analysis of apoptosis of derivatives of $CD31^+CD45^-$ cells co-culture after CX-5461 treatment.

Development • Supplementary information

E. Quantification of the frequency of apoptotic cells in derivatives of $CD31^{+}CD45^{-}$ cells co-culture treated with DMSO (n = 5), 50 nM CX-5461 (n = 6). Data are represented as mean ± SD and analyzed by unpaired two-tailed Student's t-test.

F. Representative flow cytometric analysis of cell death of derivatives of $CD31^{+}CD45^{-}$ cells co-culture after CX-5461 treatment.

G. Quantification of the frequency of dead cells in derivatives of $CD31^{+}CD45^{-}$ cells co-culture treated with DMSO (n = 5), 50 nM CX-5461 (n = 6). Data are represented as mean ± SD and analyzed by unpaired two-tailed Student's t-test.

H. Representative flow cytometric analysis of E10.5 embryonic cells co-cultured with or without OP-puro.

I. Representative flow cytometric analysis of OP-$puro^{+/high}$ cells in E10.5 caudal half AECs ($CD41^{-}CD43^{-}CD45^{-}CD31^{+}CD201^{-}Kit^{-}CD44^{+}$), HECs ($CD41^{-}CD43^{-}CD45^{-}CD31^{+}CD201^{+}Kit^{+}CD44^{+}$) and E11.0 AGM T1 pre-HSCs ($CD31^{+}CD45^{-}CD41^{low}$).

J. Quantification of the frequency of OP-$puro^{+/high}$ cells in E10.5 caudal half AECs ($CD41^{-}CD43^{-}CD45^{-}CD31^{+}CD201^{-}Kit^{-}CD44^{+}$), HECs ($CD41^{-}CD43^{-}CD45^{-}CD31^{+}CD201^{+}Kit^{+}CD44^{+}$) and E11.0 AGM T1 pre-HSCs ($CD31^{+}CD45^{-}CD41^{low}$).

K. Representative flow cytometric analysis of mean fluorescence intensity (MFI) of OP-puro signal in E10.5 caudal half AECs ($CD41^{-}CD43^{-}CD45^{-}CD31^{+}CD201^{-}Kit^{-}CD44^{+}$), HECs ($CD41^{-}CD43^{-}CD45^{-}CD31^{+}CD201^{+}Kit^{+}CD44^{+}$) and E11.0 AGM T1 pre-HSCs ($CD31^{+}CD45^{-}CD41^{low}$).

L. Quantification of the MFI of OP-puro signal in E10.5 caudal half AECs ($CD41^{-}CD43^{-}CD45^{-}CD31^{+}CD201^{-}Kit^{-}CD44^{+}$), HECs ($CD41^{-}CD43^{-}CD45^{-}CD31^{+}CD201^{+}Kit^{+}CD44^{+}$) and E11.0 AGM T1 pre-HSCs ($CD31^{+}CD45^{-}CD41^{low}$).

Development • Supplementary information

Supplemental Figure 4

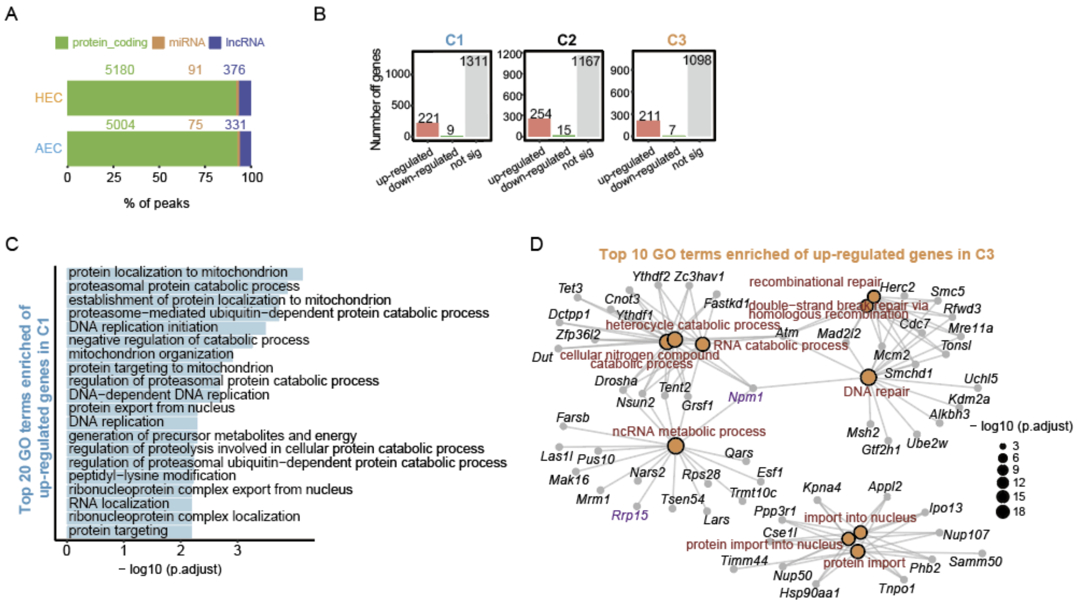

Fig. S4. Runx1 pre-binds RiBi-related genes locus and mediated their expression.

A. Bar plots displaying the annotation of the Runx1-binding peaks identified by ChIP-seq data.

B. Quantification of the number of DEGs in C1, C2, and C3.

C. Top 20 enriched Gene Ontology biological process (GO: BP) terms by using up-regulated genes in C1.

D. Network showing top 10 enriched GO terms associated with corresponding genes, generated using up-regulated genes in C3.

Development • Supplementary information

Table S1. Immunophenotype, location and developmental stages of cell populations.

Available for download at
https://journals.biologists.com/dev/article-lookup/doi/10.1242/dev.202875#supplementary-data

Table S2. Genes with RUNX1 binding in AECs and HECs.

Available for download at
https://journals.biologists.com/dev/article-lookup/doi/10.1242/dev.202875#supplementary-data
